# Sustainable Development Competencies among More than 1100 Certified Physical Education and Health Teachers in Sweden

**DOI:** 10.3390/ijerph192315914

**Published:** 2022-11-29

**Authors:** Andreas Fröberg, Petter Wiklander, Suzanne Lundvall

**Affiliations:** 1 Department of Food and Nutrition, and Sport Science, University of Gothenburg, Läroverksgatan 5, P.O. Box 300, 405 30 Gothenburg, Sweden; 2 Department of Sport, Food and Natural Sciences, Western Norway University of Applied Sciences, Høgskulen på Vestlandet Campus Sogndal, Postboks 7030, 5020 Bergen, Norway

**Keywords:** 2030 agenda, education for sustainable development, physical education, sustainable development goals

## Abstract

School physical education and health (PEH) may not only be an important cornerstone to the holistic development of students but may also contribute to the sustainable development (SD) agenda. Although PEH may have unique characteristics that can contribute to the SD agenda, most research to date has been theoretical. The overall aim of this study was to explore the sustainable development competencies among physical education and health (PEH) teachers in Sweden. An online questionnaire was used to collect data about background and SD competencies. SD competencies was collected through the use of the Physical Education Scale for Sustainable Development in Future Teachers (PESD-FT). Of the 1153 participants, 31% reported being males, and 48% of the participants reported teaching PEH when completing the questionnaire. The median SD competencies score for all the participants was 105 (range: 18–144) out of 144. Virtually no differences were observed across the groups of participants. A stronger correlation was observed between SD competencies vs. long-time interests in health and health issues (r_s_ = 0.343) than for long experience of participating in organized sports (r_s_ = 0.173). In the total sample, 26% reported having taught about SD in PEH, such as using outdoor education, interdisciplinary projects, picking, and sorting waste, as well as paying attention to material issues. Among those who reported teaching PEH when completing the questionnaire, 70% perceived that they are in great need of professional development education in the area of SD. In conclusion, SD competencies were higher for the PESD-FT items that concerned the social dimension of SD compared to the economic and environmental dimensions. Relatively few teachers had taught about SD in PEH, and the majority perceive that they are in great need of professional development education in the area of SD. Future studies are required to understand more of what types of competencies practicing PEH teachers, and PEH teacher education programs, are lacking to fulfil the call for a contribution to the SD agenda.

## 1. Introduction

The global challenges that humanity faces, including inequality and climate change, are at the point of departure in the sustainable development (SD) agenda. Perhaps the most broad and ambitious action plan for SD is the 2030 agenda that was introduced by the United Nation in 2015 [1]. The 2030 agenda comprises 17 intertwined SD goals (SDGs) and 169 associated targets that cover the social, economic, and environmental dimensions of SD. As one cornerstone, education has the potential to empower people with SD competences to make responsible decisions in the pursuit of a just society in present and future generations, and to fulfil the 2030 agenda. Some critical SD competences may be systems thinking, strategic thinking, critical thinking, collaboration, and problem-solving [2].

School physical education and health (PEH) may not only be an important cornerstone to the holistic development of students but may also contribute to the sustainable development (SD) agenda. Even though Lake et al. discussed issues around sustainability already at the beginning of the 2000s, this perspective has received limited attention in research about PEH [3]. Recently, however, there has been a growing interest in exploring links between PEH and the SD agenda, including links to the 2030 agenda and the SD goals (SDG). This may be important because the unique subject characteristics of PEH, such as movement education and health, can have distinct contributions to the SD agenda.

Some recent research suggests that PEH may already be implementing content to promote SD competences without making explicit references to the SD agenda [4,5]. In relation to the 2030 agenda and the SDGs, however, relatively little is currently known about the distinct role of PEH, and how SD can be understood, framed, and integrated in PEH [6]. Although not all may be relevant, targets from several SDGs could be addressed through PEH, including good health and well-being, gender equality, and reduced inequalities, together representing the social, economic, and environmental dimensions of SD [4,7,8]. Incorporate the SD perspective in the field of PEH may involve not only critically reviewing and revising the curricula, but also to reconsider the learning perspectives and orientation towards health and well-being in relation to oneself and others [8]. Importantly, rather than additions of content to an already overcrowded curriculum, SD perspectives in the field of PEH should be interpreted as an overarching teaching approach and tie to core areas of the subject, such as physical activity, movement education, and health and well-being [4,5]. This may, however, necessitate novel teaching approaches, and professional development education [4].

Although PEH may have unique characteristics that can contribute to the SD agenda, most research to date has been theoretical. Little is therefore currently known about SD from the perspectives of PEH teachers and students. One previous study from Spain explored SD awareness among 203 PEH teachers. The authors found that these PEH teachers generally had a high SD awareness, except for behavior in the economic dimension. In addition, they observed higher scores among female teachers for all the variables assessed, except for knowledge and behavior in the social dimension [9]. Another study among undergraduate students who were majoring in PEH indicated that they generally had a high self-perception in SD competencies [10]. Another finding of the study was that sport-competitive experience was related to higher competencies for the social dimension of SD [10]. Together, previous studies appear to report mixed findings for SD competences depending on sex, age, and previous experiences. However, more studies would increase our knowledge regarding SD competencies among PEH teachers. The present study adds to the literature by exploring SD competencies among certified PEH teachers in Sweden.

### Context and Theoretical Background

The participants of the present study are PEH teachers in Sweden. In Sweden, PEH cover areas such as movement, health and lifestyle, and outdoor life and activities (outdoor visits). The core content includes movement activities, both indoors and outdoors, different aspects of health and training methods, and safety and consideration in connection with various activities [11].

In the PEH syllabi in Sweden, there are no explicit statements related to SD. In the supporting commentary material, that intends to provide a broader and deeper understanding of the selections and positions behind the broad and often vaguely defined content in the PEH syllabi, there are some explicit statements made in relation to outdoor life and nature and ergonomics [12]. The Swedish National Agency for Education’s (SNAE) curriculum for compulsory school, including grade levels 1 to 9, includes explicit statements related to SD that are common to the school in general and all subjects. One example of such statements is that “teaching should illuminate how the functions of society and our ways of living and working can best be adapted to create sustainable development” [13] (p. 8).

As is the case internationally, few studies from Sweden have focused on SD in the context of PEH. Previous studies which examined teachers’ and students’ perceptions of PEH suggest, however, that much of the content taught in PEH in Sweden resembles organized sport activities, that being physically active is an important part of the subject, and that teachers not always communicate the aims and learning goals of lessons [14,15,16,17,18]. Despite this focus on sport and physical activity, we argued that PEH, both in Sweden and other countries, may have unique characteristics that can contribute to the SD agenda. Currently, however, we lack empirical studies with data collected from PEH teachers in Sweden. More specifically, we lack information about SD competencies among PEH, if and how they teach SD, and if they perceive themselves as having the knowledge and expertise necessary to do so. Some previous studies among Swedish upper secondary school teachers within subjects such as science, social science, language, and vocational and esthetical–practical subjects, suggest that they are influenced by their subject traditions, and lack inspiring examples and the knowledge and expertise necessary to teach their pupils about SD [19]. It has also been shown that teachers from different subjects emphasize different dimensions of SD, such as science teachers emphasize the environmental dimension, whereas social science teachers emphasize the social dimensions of SD [20]. Additionally, more than 70% of the teachers perceive themselves as being in need of professional development education in the area of SD [20]. However, no study to date has specifically focused on PEH teachers.

Against this theoretical background, we add to the literature by addressing some of the above research gaps. The present study will shed light on SD from the perspectives of PEH teachers. The overall aim is to explore SD competencies among certified PEH teachers in Sweden.

## 2. Materials and Methods

The participants of the present study were certified PEH teachers in Sweden. Data were collected using an online questionnaire that had two sections: one section to collect data about background, and one section to collect data for SD competencies in relation to PEH.

### 2.1. Participants

The participants were recruited through a digital register with E-mail addresses to certified (diplomas of certification) teachers provided by the SNAE. In Sweden, teacher certifications are granted to those who meet certain national requirements for skills deemed important for working as a teacher in the school system. The teacher certifications specify the subjects and grade levels a teacher is qualified to teach. This includes certification to teach PEH in different combinations of grade levels within the following school forms: preschool, compulsory school (grade levels 1 to 9), upper secondary school (grade levels 10 to 12), special education, and adult education. SNAE issues these certifications and keep records of all the certified teachers in Sweden.

In total, 2078 certified PEH teachers answered the questionnaire. For the aim of this study, however, we focus on those 1153 participants that had teacher certification for preschool and/or compulsory school (grade levels 1 to 9). Of these, 31% (n = 358) reported being males and 69% (n = 791) were females (0.3%, n = 4 reported other), and the mean (±SD) age among the participants was 50 (±11) years. Most participants reported that they graduated after 2000 (53%), and 81% had PEH certificates valid for preschool to grade 3, whereas 68% had certificates for grade levels 4 to 6, and 23% for grade levels 7 to 9. In total, about half (48%) of the participants reported teaching PEH when completing the questionnaire. These participants reported teaching pre-school to grade level 3 (58%), grade levels 4 to 6 (49%), and grade levels 7 to 9 (23%), and most were employed in public school (89%). See Table 1 for more background information.

The study design and its procedures were in accordance with the ethical standards of the Declaration of Helsinki. Prior to the completion of the questionnaire, the participants receive information regarding the aim and procedure of the study. Contact information were also addressed if the participants had additional questions related to the study, its design, and procedure. Participation was voluntary and indirect informed consent was obtained, meaning that the participants agreed to the terms and conditions of the study when they had filled out and submitted the questionnaire.

### 2.2. Online Questionnaire

Webropol 3.0 survey and reporting tool (https://webropol.com/ (accessed on 31 March 2022)) was used to send the questionnaire to the E-mail addresses in June 2022. Two reminders were sent in August and September 2022, and the questionnaire was closed in October 2022.

#### 2.2.1. Background Information

We asked the participants to provide information on their sex (male, female, or other) and year of birth. We also asked about their graduation year and for which grade levels their PEH certifications were valid. Furthermore, we asked whether they were employed as a PEH teacher when completing the questionnaire, in what grade levels, and in what type of school organization (public, independent, or governmental), as well as how many years they had been employed as a PEH teacher.

Moreover, we asked the participants to indicate whether they had long previous experience of participating in organized sports, exercise, outdoor life, and dance. An example of such a statement was “I have long experience of participating in organised sports”. In addition, we asked the participants to indicate whether they for a long time had been interested in health, health issues, and everyday physical activity. An example of such statement was “I have been interested in health and health issues for a long time”. Each of these statements was responded to using an eight-point Likert scale ranging from 1 (strongly disagree) to 8 (strongly agree).

Furthermore, the participants were asked whether they ever had taught about SD in PEH. A follow-up question with open response alternative was provided, and we asked the participants to give examples of working areas, themes, activities, or projects related to SD. We also included the statement “I am in great need of professional development education in the area of sustainable development” that was answered using an eight-point Likert scale ranging from 1 (strongly disagree) to 8 (strongly agree) were used.

Finally, we also included two statements about SD in relation to the (Swedish) PEH syllabus. These two statements were: “PEH has good conditions for working with sustainability issues based on current syllabus”, and “the overall aim of PEH relates well to abilities and behaviors that promote sustainable development”. Similar to the above, the participants answered the two statements using an eight-point Likert scale ranging from 1 (strongly disagree) to 8 (strongly agree).

#### 2.2.2. Sustainable Development Competencies

To explore SD competencies among PEH teachers, we used the Physical Education Scale for Sustainable Development in Future Teachers (PESD-FT) that was developed by Baena-Morales et al. [21]. When designing the PESD-FT, they considered 24 of the 169 targets that constitutes the SDGs that they believe could be worked on in PEH [7]. Ten experts were involved in the validations process, and a validation study among 340 physical activity and sport science students demonstrated that the PESD-FT had a high reliability (0.949) and validity criteria (0.929). Furthermore, the results from the principal component factor analysis indicate that the PESD-FT comprises three factors, which match with the social, economic, and environmental dimensions of SD [21].

The original PESD-FT contains 20 items. Each item involves a statement that is answered using an eight-point Likert scale ranging from 1 (strongly disagree) to 8 (strongly agree). An example of a statement was “I could improve people’s physical ability through physical education”. Two items concerning sexual and reproductive health and road safety were removed from the questionnaire as these areas are not represented in the Swedish PEH syllabus, leaving a total of 18 items. Of these, 6 items reflected the social dimension of SD (items 1–4 and 6–7), whereas 6 reflected the economic (items 5 and 9–13), and another 6 reflected the environmental (items 8 and 14–18) dimension. Before the items, the participants were presented with the following description of the concept SD: “sustainable development may be defined as development that meets the needs of the present without compromising the ability of future generations to meet their own needs”.

Prior to the data collection, we translated the PESD-FT items from English to Swedish. The translated questionnaire was pilot tested by a group of four certified PEH teachers who provided open-ended feedback for its simplicity and feasibility. The open-ended feedback process revealed that the items and response alternatives were clear and understandable.

### 2.3. Processing and Analysing Data

The collected background information was used to create a different age group, as well as groups based on the participants’ graduation year and number of years teaching PEH. We pooled the original eight-point Likert scale that was used when collecting background information, as well as for each statement in the PESD-FT, into the following four categories: disagreed (1–2), somewhat disagreed (3–4), somewhat agreed (5–6), and agreed (7–8). We created an SD competence index (SDC-I) by summarizing the total score for all the 18 items. The minimum score was 18 and the maximum 144, and the higher the SDC-I score, the higher the SD competence. We also created a score for each of the three dimensions of SD, namely the social, economic, and environmental dimensions (minimum and maximum scores were 6 and 48, respectively).

Descriptive statistics—both the means (±SD) and medians (range)—were calculated and reported for the continuous variables. The test of normality and visual examination of the histograms indicated that the distribution for the SDC-I score was non-normal. Therefore, the non-parametric Kruskal–Wallis and Mann–Whitney U-test were used to explore differences in the SDC-I score across the different groups of participants (sensitivity test using parametric statistics indicated the same results). Furthermore, proportions (%) were calculated for the categorial variables. The chi-square (χ2) test was used to explore differences in the distribution of proportions for each of the 18 PESD-FT items across different groups of participants.

Using Spearman’s rank-order correlation (r_s_), we also explored correlations between the SDC-I score vs. experiences of participating in organized sports, exercise, outdoor life, and dance, and long-time interests in health, health issues, and everyday physical activity. In these analyses, we kept the original eight-point Likert scale used for the statements related to previous experiences and long-time interests. The correlation coefficients were interpreted using the following values as a rule of thumb: 0.01–0.30 (small), 0.30–0.50 (moderate), 0.50–0.70 (large), 0.70–0.90 (very large), and 0.90–1.00 (extremely large) [22]. All analyses were performed with IBM SPSS Statistics for Windows, Version 29.0. (IBM Corp. in Armonk, New York, USA), and the alpha-level was set to 5% (*p* < 0.05).

Finally, we thoroughly read the examples of working areas, themes, activities, or projects related to SD, as stated by the participants. We then qualitatively analyzed and categories the examples into nine broad themes. Although these themes were somewhat overlapping, they captured the overall content of the examples provided by the participants.

## 3. Results

The mean (±SD) SDC-I score for all the participants was 104 (±23), whereas the median was 105 (range: 18–144) (see Table 1). The pooled mean and median values for the six items that concerned the social dimension of SD were 39.6 (±7.5) and 41 (6–48), respectively. For the economic dimension, the pooled mean SDC-I score was 32.5 (±9.0), whereas the median was 33 (range: 6–48). The corresponding figures for the environmental dimension was 32.2 (±9.6) and 32 (range: 6–48), respectively. Moreover, the mean (±SD) values for the three dimensions were as follows: 6.6 (±1.2) for the social dimension, 5.4 (±1.5) for the economic dimension, and 5.4 (±1.6) for the environmental dimension. Female PEH teachers had higher median values for items that concerned the environmental dimension (females: 6, males: 5, *p* = 0.003), but no differences were observed for the social and economic dimensions (both *p* > 0.05).

Table 1 presents the median values for SDC-I scores across different groups. There were no differences in the SDC-I score between males and females (*p* = 0.185), age groups (*p* = 0.609), groups of graduation year (*p* = 0.413), and for how long their PEH certificate has been valid (grade levels) (all *p* > 0.05). Similarly, no differences in the SDC-I scores were observed between those who reported teaching PEH when completing the questionnaire and those who did not (*p* = 0.246). However, those who reported teaching PEH in grade levels 4 to 6 had a significantly higher median SDC-I score compared to those who did not (108 (45–144) vs. 103 (43–144), *p* = 0.028). Furthermore, no differences were observed between the different types of school (public, independent, or governmental) (all *p* > 0.05) and between the number of years teaching PEH (*p* = 0.168).

**Figure 1:**
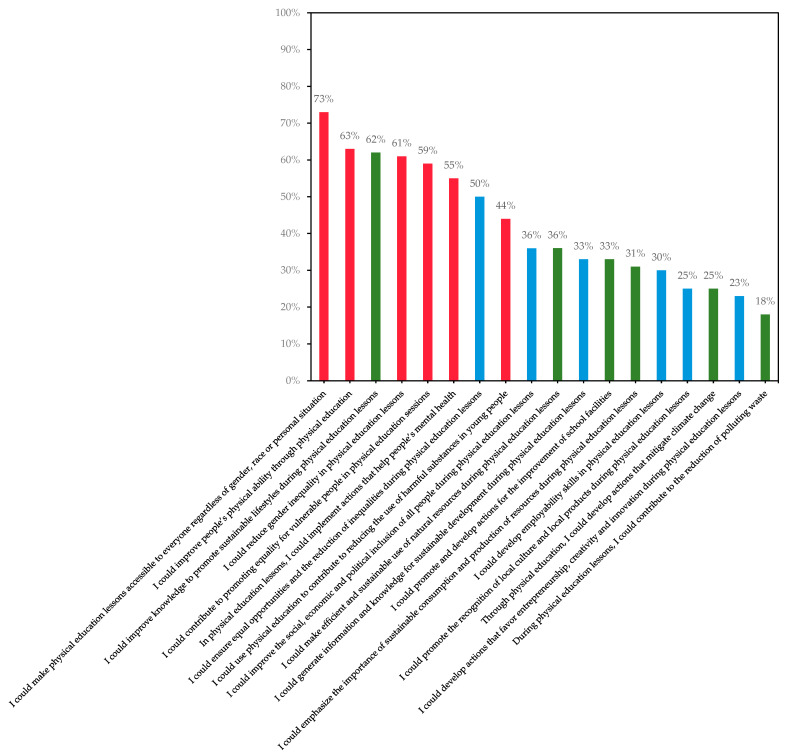
The proportions of participants (n = 1153) who agreed to each of the 18 items. Note: the items are in descending order from left to right. The colors represent different dimensions of SD: social dimension (red), the economic dimension (blue), and environmental dimension (green).

**Figure 2:**
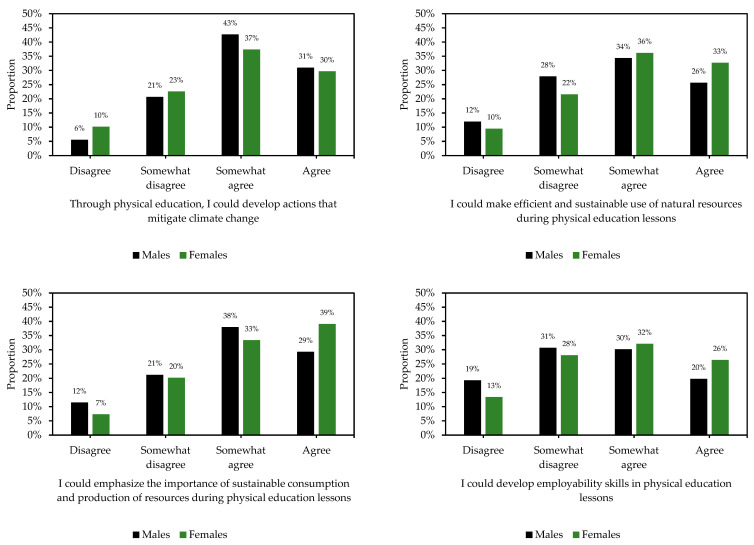
Distribution of proportions for the four items that differed between males and females (see Table 2) (total sample: n = 1153).

Table 2 provides an overview of the distribution of proportions for each of the 18 items in the PESD-FT. The mean (±SD) and median (range) values for each item is also presented in Table 2. Figure 1 presents the proportion of participants who agreed to each of the 18 items. Most participants agreed with the items about making lessons accessible to everyone regardless of gender, race, or personal situation (73%), followed by the items about improving people’s physical ability (63%), and improving knowledge to promote sustainable lifestyles (62%). In contrast, few agreed to the items about contributing to the reduction in polluting waste (18%), and the development of actions that favor entrepreneurship, creativity, and innovation (23%). Furthermore, there were no differences between males and females for 14 of the 18 items. Figure 2 shows the distribution of proportions across the four items that differed between males and females. A higher proportion of males somewhat agreed or agreed to the statement concerning the development of actions that mitigate climate change compared to females (the environmental dimension). In contrast, a higher proportion of females somewhat agreed or agreed to the other three statements that concerned the efficient and sustainable use of natural resources, emphasizing the importance of sustainable consumption and the production of resources (both the environmental dimension), as well as the development of employability skills in physical education lessons (the economic dimension).

Table 3 shows the correlations between the SDC-I score vs. the experiences of participating in organized sports, exercise, outdoor life, and dance, and long-time interests in health, health issues, and everyday physical activity. In both the total sample, and among males and females, there were significant small correlations between the SDC-I score vs. previous experiences of participating in organized sport, exercise, outdoor life, dance, and everyday physical activity (r_s_ = from 0.167 to 0.275, all *p* < 0.001). There were also significant moderate correlations between the SDC-I score vs. long interests in health and health issues (r_s_ = from 0.318 to 0.350, all *p* < 0.001). In the total sample, the smallest correlation was observed for the SDC-I score vs. long experience of participating in organized sports (r_s_ = 0.173, *p* < 0.001) (see Table 3). The strongest correlations (although moderate) were observed between the SDC-I score vs. long-time interests in health and health issues (r_s_ = 0.343, *p* < 0.001).

Moreover, in the total sample, 26% reported having taught their pupils about SD in PEH. Of those who reported teaching PEH when completing the questionnaire, 33% had taught SD in PEH, whereas the corresponding figure for those who did not was 19%. Table 4 presents the themes that together captured the examples of working areas, activities, or projects related to SD, as stated by the participants.

**Figure 3:**
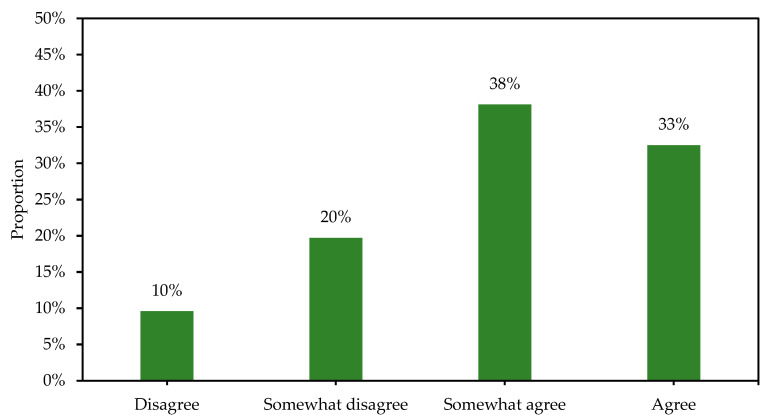
Distribution of proportions for the statement “I am in great need of professional development education in the area of sustainable development” for those participants who reported teaching PEH when completing the questionnaire (total sample: n = 553).

**Figure 4:**
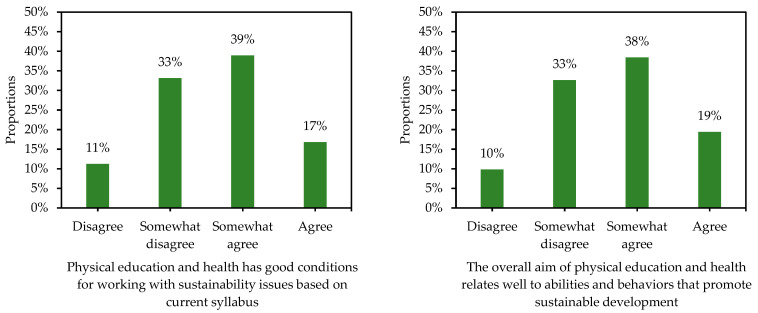
Distribution of proportions for the two statements about SD in relation to the PEH syllabus for those participants who reported teaching PEH when completing the questionnaire (total sample: both n = 553).

Among those who reported teaching PEH when completing the questionnaire, about 70% somewhat agreed or agreed to the statement “I am in great need of professional development education in the area of sustainable development” (Figure 3). Finally, about half of those who reported teaching PEH somewhat agreed or agreed to the two statements “PEH has good conditions for working with sustainability issues based on current syllabus” (56%), and “the overall aim of PEH relates well to abilities and behaviors that promote sustainable development” (57%) (see Figure 4).

## 4. Discussion

The aim of the present study was to explore SD competencies among certified PEH teachers in Sweden. In our sample of more than 1100 certified PEH teachers, the SDC-I score was 105 out of 144, and the score was higher for the PESD-FT items that concerned the social dimension of SD compared to the economic and environmental dimensions. Among the items that concerned the social dimensions of SD, most participants agreed with the items about making PEH lessons accessible to everyone regardless of gender, race, or personal situation, and that PEH can be used to improve people’s physical ability, as well as to improve knowledge to promote sustainable lifestyles. Compared to a previous study among undergraduate students who were majoring in PEH in Spain, the mean values for the pooled items that concerned the social dimension of SD was similar (a mean of 6.6 in both studies). However, the mean values for the pooled items concerning economic and environmental dimensions were somewhat lower in our sample. For both dimensions, the mean was 5.4 in this study, and 6.3 in the previous study [10].

Furthermore, our findings were somewhat consistent, and we observed a few differences across the groups of participants. However, some differences were observed between male and female PET teachers. Female teachers had higher mean values for the PESD-FT items that concerned the environmental dimension. When considering specific items, a higher proportion of male teachers somewhat agreed and agreed to one item that concerned the environmental dimension, whereas the opposite was shown for three other items that concerned the environmental (n = 2 items) and economic (n = 1 item) dimensions. Previous research among PEH teacher students and in-service PEH teachers show somewhat mixed results for sex differences, with both a higher awareness among females [9] and no differences between males and females [10].

In relation to previous experiences and long-time interest, our analyses showed the strongest correlation (although moderate) between the SDC-I score and long-time interests in health and health issues. Although the correlation coefficient was modest (r_s_ = 0.343), it was higher compared to, for example, long experience of participating in organized sports (r_s_ = 0.173). This finding is interesting as it suggests that the perceived SD competencies among PEH teachers may differ depending on previous experiences and how interested they were. The correlation coefficient for organized sports, however, is similar to a previous study that analyzed the correlations between the pooled items for the social dimension of SD and sport-competitive experience (r_s_ = 0.158) [10]. Future studies should continue exploring the SD competencies among PEH teachers with different experience of participating in various kind of activities, including organized sports and exercise, as well as other long-time interests in health, health issues, and everyday physical activity.

It has previously been suggested that PEH may already Involve content to promote SD competences without making explicit references to the SD agenda [4,5]. In this sample of PEH teachers, about a third of those who reported teaching PEH when completing the questionnaire stated that they had taught their pupils about SD. The examples provided by the participants suggested that some common strategies to teach about SD included the usage of outdoor education, picking and sorting waste, and by paying attention to material issues, such as informing about the possibility of the importance of borrowing, exchanging, and reusing materials. Another common strategy was interdisciplinary projects to cooperate with other school subjects, such as home and consumer studies, biology, and geography. Examples similar to these are worth paying attention to in future studies. For example, qualitative studies could explore how PEH teacher make sense of these types of strategies, and in what way they perceive that the working areas, activities, or projects related to the SD agenda, such as SD dimensions or even specific SDGs or the associated targets.

Given the fact that two thirds reported that they had not taught about SD, however, one important finding of this study is that 70% of the participants perceive that they are in great need of professional development education in the area of SD. Professional development education should be designed to empower PEH teachers to teach about SD, and how to include SD as an overall teaching approach. Ultimately, such education should be subject specific and pay attention to the unique characteristics of PEH that can have distinct contributions to the SD agenda, such as movement education and health. It should also be mentioned that, if any changes in relation to education for SD are to take place in school, teacher education is one critical point of departure, which has previously been an underutilized resource. In many ways, teacher education should contribute to the development of the knowledge, skills, and abilities needed for tomorrow’s teachers to become active change agents to bring about changes in the school curriculum. We have in a previous study analyzed almost 500 learning outcomes retrieved from course syllabi at Swedish PEH teacher education institutions and found that few of these learning outcomes explicitly related to SD perspectives. As such, we concluded that PEH teacher educators should reflect on what it can mean to include SD perspectives in PEH teacher education courses [23]. While professional development education may be needed, it is important to stress that SD and sustainability-oriented learning is not only a concern of PEH but something that is common to all subjects and the school in general.

Moreover, only half of those who reported teaching PEH somewhat agreed or agreed that PEH has good conditions for working with SD based on the current syllabus and the overall aim of the subject. From our point-of-view, this finding was somewhat expected. In a previous study, we explore how SD, and sustainability-oriented learning, were reflected in the PEH syllabus in Sweden. We found no explicit statements related to SD, and we concluded that there is a gap in the PEH syllabus in terms of addressing SD and sustainability-oriented learning [12].

In this first study, to explore SD competencies among certified PEH teachers in Sweden, one strength is the large sample size. Another strength is that the data were collected with a questionnaire that has shown to have a high reliability and validity [21]. A common limitation is that questionnaires may be related to social desirability, which may introduce error and bias. Hypothetically, such social desirability may particularly be observed in research dealing with SD, yet some research suggests that the effect of social desirability on environmentally relevant behaviors, intentions, and attitudes may be small [24]. The issue of social desirability can, however, not be ruled out. Similarly, those who responded to the questionnaire may also be those who are interested in SD in general, and perhaps SD in the context of PEH in particular. These limitations should be taken into account when interpreting the findings of the present study.

## 5. Conclusions

In conclusion, SD competencies were higher for the PESD-FT items that concerned the social dimension of SD compared to the economic and environmental dimensions. Relatively few teachers had taught their pupils about SD in PEH, and the majority perceive that they are in great need of professional development education in the area of SD. This touches upon the role of PEH teacher education, and what kind of knowledges that are supposed to be covered and found legitimate to secure SD competencies. Future studies are required to understand more of what types of competencies PEH teachers, and PEH teacher education programs, are lacking to fulfil the call for a contribution to the SD agenda.

## Figures and Tables

**Table 1:** Study participant characteristics and SDC-I score across different groups of participants (total sample: n = 1153).

		SDC-I Score **		
	Proportion (%)	Mean (±SD)	Median (Min–Max)	*p*
Sex				0.185
Males	31%	103.1 (±22.2)	103 (18–144)	
Females	69%	104.6 (±23.6)	106 (18–144)	
Age group				0.609
20–29 years	4%	105.8 (±17.0)	102 (69–144)	
30–39 years	14%	104.7 (±20.7)	104 (57–144)	
40–49 years	30%	105.9 (±21.0)	108 (48–144)	
50–59 years	33%	104.1 (±23.2)	105 (18–144)	
60+ years	19%	101.0 (±28.3)	104 (18–144)	
Graduation year				0.413
1970–1979	5%	105.1 (±30.1)	109 (18–144)	
1980–1989	13%	101.1 (±26.3)	101 (18–144)	
1990–1999	29%	104.1 (±23.0)	107 (18–144)	
2000–2009	28%	103.6 (±23.1)	105 (18–144)	
2010–2022	25%	106.4 (±19.7)	106 (54–144)	
Grade levels PEH certificate valid *				
Pre-school to grade level 3	81%	104.8 (±22.3)	105 (18–144)	0.186
Grade levels 4 to 6	68%	104.7 (±22.6)	106 (18–144)	0.312
Grade levels 7 to 9	23%	104.8 (±21.3)	106 (34–144)	0.817
Taught PEH when answering the questionnaire				0.246
Yes	48%	105.6 (±20.9)	106 (43–144)	
No	52%	102.9 (±25.0)	104 (18–144)	
Grade levels teaching PEH *				
Pre-school to grade level 3	59%	105.7 (±20.5)	106 (45–144)	0.948
Grade levels 4 to 6	**49%**	**107.6 (±20.1)**	**108 (45–144)**	**0.028**
Grade levels 7 to 9	23%	105.7 (±22.0)	107 (43–144)	0.828
Type of school *				
Public	89%	104.0 (±23.1)	105 (18–144)	0.382
Independent	10%	104.3 (±24.8)	105 (18–144)	0.688
Governmental	3%	108.7 (±21.2)	105 (60–144)	0.312
Number of years teaching PEH				0.168
1–9 years	54%	102.6 (±24.1)	103 (18–144)	
10–19 years	27%	105.5 (±22.5)	106 (18–144)	
20–29 years	14%	106.7 (±19.7)	106 (57–144)	
30+ years	5%	107.8 (±24.4)	109 (59–144)	

Abbreviation: PEH, physical education and health; SDC-I, sustainable development competence index. Note: the Mann–Whitney U-test and Kruskal–Wallis were used to analyze data. * The participants could provide multiple response options, which mean that (i) the absolute number of responses exceeded the number of participants; (ii) the proportions (%) do not add up to 100%. Therefore, the analyses for these variables were conducted separately for each group (e.g., for the variable grade levels PEH certificate valid, the group pre-school to grade level 3 was treated as yes vs. no). ** The SDC-I was created by summarizing the total score for all the 18 items (minimum score is 18 and maximum score is 144). Significant differences between groups of participants indicated as bold.

**Table 2:** Distribution of proportions, mean and median values for each of the 18 items in the PESD-FT in the total sample (n = 1153).

		Proportions (%)					Average		
Dimension	Item	Disagreed	Somewhat Disagreed	Somewhat Agreed	Agreed	*p*-Value between Sex	Mean (±SD)	Median (Min–Max)	*p*-Value between Sex
Social	I could improve people’s physical ability through physical education.	2%	7%	28%	63%	0.145	6.8 (±1.5)	7 (1–8)	0.078
Social	In physical education lessons, I could implement actions that help people’s mental health.	2%	9%	34%	55%	0.197	6.6 (±1.6)	7 (1–8)	0.723
Social	I could use physical education to contribute to reducing the use of harmful substances in young people.	5%	16%	35%	44%	0.196	6.0 (±1.9)	6 (1–8)	0.943
Social	I could make physical education lessons accessible to everyone regardless of gender, race, or personal situation.	2%	4%	20%	73%	0.839	7.0 (±1.5)	8 (1–8)	0.850
Economic	I could develop employability skills in physical education lessons.	**9%**	**22%**	**39%**	**30%**	**0.038**	**5.4 (±2.0)**	**5 (1–8)**	**0.027**
Social	I could reduce gender inequality in physical education lessons.	2%	7%	30%	61%	0.337	6.7 (±1.6)	7 (1–8)	0.644
Social	I could contribute to promoting equality for vulnerable people in physical education sessions.	2%	8%	32%	59%	0.102	6.6 (±1.5)	7 (1–8)	0.486
Environmental	I could improve knowledge to promote sustainable lifestyles during physical education lessons.	1%	6%	31%	62%	0.344	6.8 (±1.4)	7 (1–8)	0.266
Economic	I could promote and develop actions for the improvement of school facilities.	11%	21%	35%	33%	0.177	5.4 (±2.1)	6 (1–8)	0.344
Economic	I could develop actions that favor entrepreneurship, creativity, and innovation during physical education lessons.	12%	26%	38%	23%	0.402	5.0 (±2.0)	5 (1–8)	0.402
Economic	I could promote the recognition of local culture and local products during physical education lessons.	14%	25%	36%	25%	0.067	5.0 (±2.1)	5 (1–8)	0.144
Economic	I could improve the social, economic, and political inclusion of all people during physical education lessons.	10%	21%	33%	36%	0.838	5.5 (±2.0)	6 (1–8)	0.197
Economic	I could ensure equal opportunities and the reduction in inequalities during physical education lessons.	4%	13%	34%	50%	0.215	6.2 (±1.7)	6 (1–8)	0.177
Environmental	I could emphasize the importance of sustainable consumption and production of resources during physical education lessons.	**10%**	**24%**	**36%**	**31%**	**0.019**	**5.3 (±2.0)**	**5 (1–8)**	**0.003**
Environmental	I could make efficient and sustainable use of natural resources during physical education lessons.	**9%**	**21%**	**35%**	**36%**	**0.005**	**5.6 (±2.0)**	**6 (1–8)**	**0.004**
Environmental	During physical education lessons, I could contribute to the reduction in polluting waste.	25%	29%	28%	18%	0.235	4.3 (±2.2)	4 (16)	0.067
Environmental	I could generate information and knowledge for sustainable development during physical education lessons.	10%	22%	34%	33%	0.103	5.4 (±2.0)	**6 (1–8)**	**0.013**
Environmental	Through physical education, I could develop actions that mitigate climate change.	**15%**	**29%**	**31%**	**25%**	**0.012**	**4.8 (±2.1)**	**5 (1–8)**	**<0.001**

Note: The chi-square (χ^2^) test (categorial variables) and Mann–Whitney U-test (continuous variables) were used to analyze data. Significant differences between males and females indicated as bold.

**Table 3:** Correlation coefficient between the SDC-I score vs. experiences of participating in organized sports, exercise, outdoor life, and dance, and long-time interests in health and health issues, and everyday physical activity in the total sample and stratified by males and females (total sample: n = 1153).

	The SDC-I Score		
	Total	Males	Females
**Previous Experiences and Long-Time Interests**	**r_s_**	**r_s_**	**r_s_**
I have long experience of participating in organized sports	**0.173 (small)**	**0.220 (small)**	**0.167 (small)**
I have long experience of participating in exercise	**0.196 (small)**	**0.180 (small)**	**0.210 (small)**
I have long experience of participating in some outdoor life	**0.241 (small)**	**0.253 (small)**	**0.237 (small)**
I have long experience of participating in dance	**0.198 (small)**	**0.215 (small)**	**0.191 (small)**
I have been interested in health and health issues for a long time	**0.343 (moderate)**	**0.318 (moderate)**	**0.350 (moderate)**
I have been interested in everyday physical activity for a long time	**0.257 (small)**	**0.217 (small)**	**0.275 (small)**

Note: Analyses conducted with Spearman’s rank-order correlation. Correlation coefficients were interpreted using the following values as a rule of thumb: 0.01–0.30 (small), 0.30–0.50 (moderate), 0.50–0.70 (large), 0.70–0.90 (very large), and 0.90–1.00 (extremely large) [22]. The SDC-I was created by summarizing the total score for all the 18 items (minimum score is 18 and maximum score is 144). Previous experiences and long-time interests were answered using an eight-point Likert scale ranging from 1 (strongly disagree) to 8 (strongly agree). Significant correlations indicated as bold (all, *p* < 0.001).

**Table 4:** The themes that together captured the examples of working areas, activities, or projects related to SD, as stated by the participants.

Themes	Examples
Outdoor education	Outdoor education (“friluftsliv”) in various forms, including education about outdoor stay, right of public access, and orienteering.
Health and well-being	Activities to promote knowledge and awareness of the importance of taking care of one’s own body through different physical activities and food habits.
Interdisciplinary projects	Education about SD through different themes and projects, including whole-school projects, and interdisciplinary projects with other school subjects.
Picking and sorting waste	Activities, such as walking or jogging outdoor while picking litter, and waste and sorting and recycling garbage/waste.
Everyday physical activity and active transportation	Education about the health and environmental benefits of everyday physical activity active transportation, such as walking and cycling to/from school and leisure-time activities.
Material	Material issues, such as borrow, exchange, reuse of materials, as well as usage of alternative materials (e.g., material from nature used for the purpose of exercise).
Climate issues	Activities to promote knowledge and awareness of climate issues, such as how to take care of water resources.
Inclusion and norms	Activities to promote social inclusion and awareness of social norms.
Local environment	Activities to recognize the importance of the local environment, such as how to use the local environment to be physically active.

## Data Availability

Not applicable.
